# Early-Life Intake of an Isotonic Protein Drink Improves the Gut Microbial Profile of Piglets

**DOI:** 10.3390/ani10050879

**Published:** 2020-05-18

**Authors:** Stefan G. Buzoianu, Ava M. Firth, CallaBria Putrino, Fabio Vannucci

**Affiliations:** 1 Tonisity International, D02 TN27 Dublin, Ireland; stefan@tonisity.com; 2 California Polytechnic State University, San Luis Obispo, CA 93407, USA; callabriamia@gmail.com; 3 Veterinary Diagnostic Laboratory, University of Minnesota, St. Paul, MN 55108, USA; vannu008@umn.edu

**Keywords:** microbiota, piglets, Tonisity Px, isotonic drink, weaning, *16S rRNA* gene sequencing, *Escherichia coli*

## Abstract

**Simple Summary:**

The gut microbiota plays a key role in maintaining the overall health and homeostasis of mammals. Developing blends of nutrients that contribute to a diverse and balanced gut microbiota community thus contributes to improve pig health, particularly at weaning when rapid dietary and environmental changes occur. As shown in the present study, Tonisity Px, an isotonic protein drink that provides key nutrients for nourishing the enterocytes, increased the abundance of beneficial bacteria and decreased that of potentially-pathogenic bacteria when provided to piglets aged 2 to 8 days. This isotonic protein drink also improved the overall gut environment, both pre-weaning and post-weaning, as revealed by *16S rRNA* gene sequencing and semiquantitative *Escherichia coli* culture.

**Abstract:**

A healthy microbial community in the gut of piglets is critical to minimize the negative performance consequences associated with dietary and environmental changes that occur at weaning. Tonisity Px, an isotonic protein drink, is a potential alternative to balance the gut microbiota as it contains key ingredients for nourishing the small intestine. In the present study, 16 litters comprising 161 piglets were randomly allocated to a group to which Tonisity Px was provided from days 2 to 8 of age (TPX group) or to a control group, to which no Tonisity Px was provided. The TPX group also received Tonisity Px in the 3 days before and after weaning. At days 9, 17, and 30 of age, fecal and ileum samples were collected from piglets belonging to both groups and analyzed using *16S rRNA* gene sequencing, semiquantitative PCR of *Rotavirus* serogroups, and semiquantitative *Escherichia coli* culture. Overall, Tonisity Px increased the abundance of beneficial bacterial populations (*Lactobacillus* and *Bacteroides* species) and reduced potentially pathogenic bacterial populations (*E. coli* and *Prevotellaceae*), in both the pre-weaning and post-weaning periods.

## 1. Introduction

Pig gut colonization by microbiota is initiated at birth and shaped initially by feeding on the sow’s milk, which provides nutritional advantages to lactic acid bacteria, such as *Lactobacillus* species. Gut microbiota play a crucial role in shaping piglet growth [1] and the early colonization by these microorganisms contributes to the host’s health by promoting the establishment of gut barrier function and the maturation of the immune system [2,3]. The first few weeks of a pig’s life represent a “window of opportunity” during which the overall physiology of the gut can be conditioned towards life-long growth benefits [4,5,6]. The pre-weaning period is crucial in setting up a balanced, healthy microbiota. Various studies have shown that early microbiota changes persist through weaning and into full growth [7,8,9].

More specifically, the first week of life is the key timeframe during which a healthy microbial colonization results in adequate development of key types of immune cells and intestinal structures, as well as digestive enzymatic capacity [6,7,10]. Therefore, early-life nutritional interventions that support gut development and shape microbial colonization are warranted to reduce the weaning growth check and to improve the post-weaning performance [5,6,11].

The gut microbiota affects swine health through multiple mechanisms [12,13], as microbial communities are known to participate in the production of volatile fatty acids and vitamins, in fiber fermentation, and in the development of the immune system [14,15,16]. Furthermore, a diverse and well-balanced gut microbial community provides resistance to pathogens and improves the ability to adapt to environmental changes. As such, the gut microbiota is an attractive target for finding dietary supplements that improve animal health and well-being [17]. This is particularly relevant during early-life stages and weaning transition, which is an important time of transition and stress in animals and where the pig gut microbiota plays a pivotal role influencing the overall health and growth performance of pigs [18]. Because the pig’s gut microbiota and immune system are still developing during the weaning period, animals are notably susceptible to pathogens that might drive gut inflammation and diarrhea and, as a consequence, reduced growth performance and increased mortality [19].

It is known that micro-enteral nutrition targeting gut cells drives nutrient uptake of key molecules [20]. The isotonic protein drink Tonisity Px delivers hydration and micro-enteral nutrition to support gut development and function from as early as day 2 of life and through weaning transition. In previous studies, when administered to piglets aged 2- to 8-days-old and around weaning, Tonisity Px has been shown to modulate intestinal development by increasing villus height, reduce pre-weaning mortality, and improve post-weaning growth performance [21,22]. However, it is still unknown whether this isotonic supplement contributes to a healthy gut microbiota pre-weaning and during the weaning process.

The aim of this study was to assess if the modulatory effects of Tonisity Px are associated with changes in the gut microbial populations, and if the effects of Tonisity Px in such populations are stable over time. Principally, *16S rRNA* gene sequencing was conducted on fecal samples of piglets who were supplemented with Tonisity Px (TPX group) or not (Control group) from days 2 to 8 of age and for 3 days before and 3 days after weaning. Additionally, the incidence of *Escherichia coli* and *Rotavirus* were examined on ileal mucosal samples from the same piglets.

## 2. Materials and Methods

### 2.1. Ethics

All gilts originated from a commercial production facility in which all piglets born would have been sent for slaughter at approximately 150 days of age. No treatments were given that were outside of standard industry animal husbandry techniques. The health and welfare of all animals were monitored throughout the study by research farm staff according to the site’s standard operating protocols and veterinary recommendations.

Piglets were randomly selected for euthanasia from each litter by using a random number ranking that correlated to the piglets’ ear tag number. If the litter had eight or fewer piglets on day 9, then only one piglet per litter was taken at each time point. Otherwise, two piglets per litter were randomly selected at each time point. Euthanasia of selected piglets was carried out by sedating them with tiletamine/zolazepam (Telazol 8 mg/kg, intramuscular; Zoetis, Parsippany-Troy Hills, NJ, USA) followed by sodium pentobarbital (100 mg/kg, intravenous) upon arrival at the University of Minnesota Veterinary Diagnostic Laboratory, in accordance with the university protocols and ethics procedures. The study terminated at 14 days post-weaning, after which remaining piglets were moved to a commercial production facility for continued growth and eventual slaughter. The gilts were re-housed at a community college teaching farm.

### 2.2. Animals and Treatments

Sixteen gilts (Yorkshire × Landrace × Duroc) and their litters (161 piglets) were enrolled in the present study, which was performed at a research farm located in Minnesota, USA. Gilts were brought into the farrowing barn approximately one week before the earliest expected farrowing date. Gilts and their piglets were housed in individual farrowing crates. Average temperature in the farrowing barn was controlled at 25–27 °C.

Piglets were individually tagged and weighed within 24 h of birth. Cross-fostering was permitted within treatment groups before the administration of Tonisity Px. Piglets had *ad libitum* access to a nipple water drinker while in the farrowing crate and were given iron injections at day 3 of age. Common husbandry practices such as tail docking and castration were also carried out at that time.

A commercial lactation diet was offered to gilts in the farrowing house (Supplementary Table S1), on a farm-specific lactation feeding curve. The gilts and their litters were randomly allocated to one of two study groups, Control or supplemented with Tonisity Px (TPX), based on the order of farrowing. Control piglets received no supplementation during the pre-weaning phase, and were given creep feed from weaning up until 2 days after weaning. From days 2 to 8 of age, TPX piglets were supplemented with 500 mL of 3% Tonisity Px solution once daily in an open pan. TPX piglets also received the 3% solution at 3 and 2 days before weaning, followed by a gruel mixture of creep feed and the 3% solution from 1 day before weaning up to and including 2 days after weaning. The gruel mixture was at the ratio of 1.5 kg of 3% Tonisity Px solution with 1 kg of dry feed. At weaning (~21 days), piglets were moved to an adjacent weaning barn and segregated into pens of approximately 20 piglets per pen. Both groups had *ad libitum* access to normal feed and water after weaning. The composition of the creep feed diet has been included as Supplementary Table S2.

Morbidity and mortality were recorded daily. Diarrhea scores were recorded for each litter at days 2, 5, 9, 13, and 17. Diarrhea scores were also recorded for each pen at 2, 4, 7, 12, and 14 days post-weaning. The intake of Tonisity Px solution, gruel, and dry feed was recorded daily for each litter and pen.

Seventy piglets were randomly selected from the Control and TPX groups at day 9 (n = 13 and 12, respectively), day 17 (n = 13 and 14, respectively) and day 30 of life (n = 10 and 8, respectively). The piglets were weighed at selection and then euthanized for laboratory analyses, as described below. The actual age of each piglet at the time of sampling was arranged so that at day 9 piglets were 8- to 10-day-old. For the day 17 sampling, age varied by no more than ± 2 days from the target time point, i.e., the actual age of piglets euthanized at day 17 might have been 15 to 19 days. The day 17 time point was specifically targeted to sample the gut microbiota before the introduction of any solid feed.

### 2.3. DNA Extraction, High-Throughput Sequencing, and Bioinformatics

Fecal samples were collected from the rectum of each euthanized piglet and stored in Norgen Stool Nucleic Acid Collection and Preservation Tubes (Norgen Biotek Corporation, Thorold, ON, Canada). Tubes were immediately placed on dry ice and kept frozen at −80 °C until DNA extraction at Diversigen, Inc. (Houston, TX, USA). DNA was extracted from fecal samples using the Norgen Stool DNA Isolation Kit (Norgen Biotek Corporation), according to the manufacturer’s instructions. The v4 region of the *16S rRNA* gene was then PCR-amplified using the AccuPrime High Fidelity kit (Invitrogen, Carlsbad, CA, USA) and the primers 515F (5′ AATGATACGGCGACCACCGAGATCTACACTATGGTAATTGTGTGCCAGCMGCCGCGGTAA 3′) and 806R (5′ CAAGCAGAAGACGGCATACGAGATTCCCTTGTCTCCAGTCAGTCAGCCGGACTACHVGGGTWTCTAAT 3′) [23], which incorporated adapters for further DNA high-throughput sequencing. Amplification was performed on the On-Deck thermocycler (Hamilton Company, Reno, NV, USA) under the following conditions: initial denaturation at 95 °C for 2 min; 33 amplification cycles of 20 s at 95 °C, 45 s at 50 °C, and 90 s at 72 °C; and final extension at 72 °C for 10 min. Resulting amplicons were purified using the QIAquick PCR purification kit (Qiagen, Hilden, Germany) and quantified using Invitrogen’s Quant-iT Picogreen dsDNA assay kit. Amplicons of the right size and quality were pooled, barcoded, and cleaned for *16S rRNA* gene sequencing on the MiSeq platform (Illumina Inc., San Diego, CA, USA) using the 2x 250 bp paired-end protocol yielding pair-end reads that overlap almost completely.

The pair-end reads obtained were demultiplexed based on their unique molecular barcodes and then merged using USEARCH v 7.0.1090 [24], allowing zero mismatches and a minimum overlap of 50 bases. Merged reads were trimmed at the first base with Q5 and quality-filtered. These high-quality *16S rRNA* gene sequences were clustered into Operational Taxonomic Units (OTUs) at a similarity cutoff value of 97% using the UPARSE algorithm and mapped to an optimized version of the SILVA Database [25,26] containing only the *16S rRNA* v4 region. Abundances were recovered by mapping the demultiplexed reads to the UPARSE OTUs and a rarefied OTU table was constructed for downstream analyses of alpha-diversity (the number of species), beta-diversity (microbial composition) [27], and phylogenetic trends (relative abundance).

High-throughput sequencing data were normalized to the lowest number of reads per piglet (3306), as the rarefaction curves revealed a plateau after this point. This was done to maintain all samples in the analyses and to provide them with equal representation and weight in the analyses. The total number of OTUs (richness) and Shannon’s index were calculated to describe alpha-diversity, as a measurement of bacterial diversity within each sample. Between-sample diversity (beta-diversity) was assessed by calculating bacterial community resemblance as weighted and unweighted UniFrac metrics, using sequence-derived phylogenetic relationships to determine relatedness among OTUs, and visualized and clustered using Principal Coordinates Analysis (PCoA) with comparison (*p* values) generated by a PERMANOVA. The PERMANOVA was calculated in ATIMA by leveraging the Adonis function in the vegan package [28,29].

### 2.4. Semiquantitative *E. coli* Culture

Swabs were taken from the ileal mucosa of each piglet and then cultured at 37 °C for 18–24 h on sheep blood agar for semiquantitative *E. coli* culture, using a scale of 1+ to 4+, with 1+ indicating low growth and 4+ indicating the highest level of growth.

### 2.5. *Rotavirus* PCR

Semiquantitative reverse transcription PCR (RT-PCR) assays were performed for detecting and quantifying *Rotavirus* serogroups (RV) as previously described [30], and using an ileal segment approximately 5 cm in length. The specificity of the assay was evaluated using 61 common swine pathogens but it only detected RVA, RVB, and RVC. The sensitivity was determined using gBlock Gene Fragments (IDT, Coralville, IA, USA) containing the RVA, RVB, and RVC sequence targets. The RT-PCR had an estimated detection limit of approximately 40 RNA copies per reaction [30]. A standard curve was generated and copies per reaction were converted in cycle threshold (Ct) values for routine diagnostic purposes. Samples with Ct values < 36 were considered positive.

### 2.6. Statistical Analyses

The relative abundance of bacterial taxa was analyzed using a generalized mixed model within the GLIMMIX procedure of SAS 9.4 (SAS, Cary, NC, USA). The relative abundance of bacterial taxa was calculated as the number of reads for individual OTUs divided by the total number of reads per sample. Treatment, age, and their interaction were included as main effects in the model. Body weight at euthanasia was included as a covariate and the gilt was included as a random effect in the model.

Because data were non-normally distributed, they were first transformed using the logit transformation within the model to facilitate analysis. Data were transformed back to the original data scale for presentation of the results.

For alpha-diversity, group differences were assessed using the Kruskal–Wallis and Mann–Whitney U tests and adjusted for false discovery rate corrections using the Benjamini–Hochberg procedure. The factors included in the analysis were sampling day and treatment and treatment within sampling day. The frequency of positive results obtained for the semiquantitative *E. coli* bacterial culture and semiquantitative *Rotavirus* RT-PCR were analyzed using contingency tables and the Chi square test in SAS 9.4.

Data are presented as the mean ± standard error of the mean (SEM). For relative abundance data, the individual piglet was considered the experimental unit. Values of *p* ≤ 0.05 were considered statistically significant and 0.05 < *p* ≤ 0.10 values were considered to present a near-significant trend.

## 3. Results

### 3.1. Population Analysis

#### 3.1.1. Morbidity and Mortality

One gilt was euthanized during parturition for non-responsive dystocia. Post-mortem examination showed uterine torsion. One litter from the TPX group developed diarrhea and was treated with antibiotics at day 7. Piglets from this litter (n = 6) were therefore excluded from microbiota and *E. coli* culture/*Rotavirus* RT-PCR analyses due to the potential influence of antibiotics on the gut microbial population. No significant differences were observed for diarrhea scores between treatment groups, nor was there any significant difference in mortality between treatment groups.

#### 3.1.2. Tonisity Px and Feed Consumption

Daily consumption of Tonisity Px per litter between days 2 to 8 of age was usually 95% for six of the eight TPX litters. One litter consumed only 47% of the Tonisity Px amount offered, but this litter contained only six piglets. Another litter consumed 72% of the Tonisity Px offered, but had a diarrhea outbreak that led to the death of seven piglets and subsequent administration of injectable antibiotics at day 7 of life.

The average consumption of the Px-gruel or dry creep feed offered to each treatment group (0.23 kg/day/pig) for the immediate 3 days post-weaning was 0.220 kg/pig in the TPX group and 0.228 kg/pig in the Control group on a dry matter basis. This was not a significant difference.

### 3.2. Microbiota Richness and Diversity

**Figure 1:**
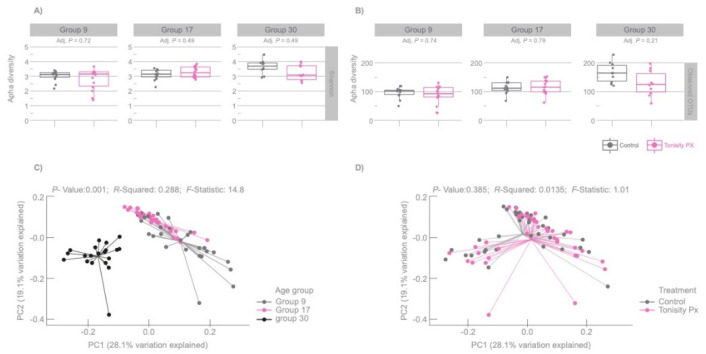
Shannon diversity index (**A**), alpha-diversity observed Operational Taxonomic Units OTUs (**B**), and beta-diversity (weighted UniFrac) of gut microbiota communities in piglets at 9, 17, and 30 days of age (**C**) and in Control and piglets supplemented with Tonisity Px (**D**).

At each sampling timepoint, Control and TPX groups had similar alpha- and beta-diversity of gut microbiota (*p* = 0.385; Figure 1). However, for both groups, age and the introduction of solid feed showed a significant effect on the gut microbiota, as communities in the pre-weaning phase (days 9 and 17) significantly differed from that in the post-weaning phase (day 30; *p* = 0.001; Figure 1). Therefore, introducing solid feed at the time of weaning clearly changed the microbiota community.

### 3.3. Effects of Tonisity Px on Gut Microbiota Abundance

As observed for microbiota diversity, significant differences in bacterial abundance at the phylum level were only detected among age groups. Overall, Bacteroidetes and Firmicutes were the dominant phyla at all ages (relative abundance > 46% and 24%, respectively). Although their relative abundances were generally higher at days 17 (52% and 31%, respectively) and 30 (46% and 35%, respectively) than at day 9 (47% and 24%), these age differences were only significant for Firmicutes (*p* < 0.05). At the family level, significant differences were also found between age groups only; *Prevotellaceae* relative abundance was the highest at all ages (*p* < 0.001) followed by Fusobacteriaceae (day 9 only) and Ruminococcaceae (days 17 and 30).

Detailed results on bacterial relative abundance presented here focus on the core genera (i.e., genera with relative abundance > 0.5%), as these are commonly found in pigs and have known roles in the gut. Table 1 presents an overview of the changes in gut microbiota communities at the genus level according to their direction of change (increase or decrease) after Tonisity Px supplementation. Further details are presented in Supplementary Table S3. Overall, there was an increase in beneficial bacteria (e.g., *Lactobacillus* and *Bacteroides*) while potentially pathogenic bacteria either decreased (e.g., *Helicobacter*) or showed no changes (e.g., *Escherichia* and *Salmonella*). For bacteria with variable roles, depending on species, sub-species, and gut conditions (e.g., *Streptococcus* and *Prevotellaceae*), increases or decreases were observed.

#### 3.3.1. Effects of Tonisity Px on Beneficial Bacteria

**Figure 2:**
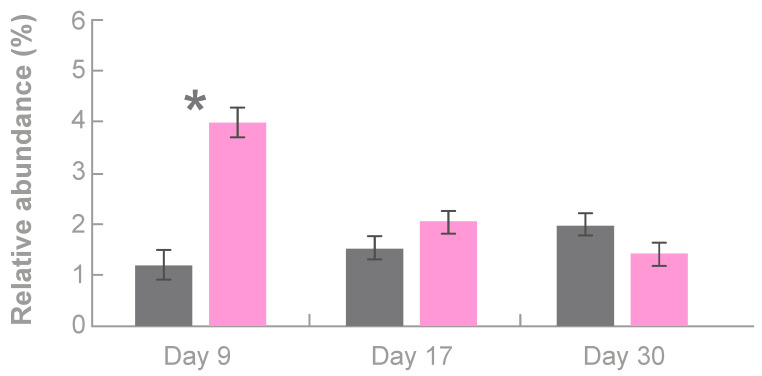
Relative abundance (%) of *Lactobacillus* species in the gut of 9-, 17-, and 30-day-old piglets in the control (grey bars) and supplemented with Tonisity Px (pink bars) groups. Bars represent LSmeans and error bars denote ± SEM. * Indicates statistical significance between treatments at *p* < 0.05.

Providing Tonisity Px at days 2 to 8 resulted in a significant 3.3-fold increase in the relative abundance of *Lactobacillus* sp. at day 9 (4.0% vs. 1.2%, respectively; SEM = 0.29; *p* = 0.0001), representing a 233% increase compared to Control piglets (Figure 2). At day 17, the relative abundance of *Lactobacillus* sp. in the TPX group was higher (2.0% vs. 1.5%; SEM = 0.23) than in the Control group, although this was not significantly different.

**Figure 3:**
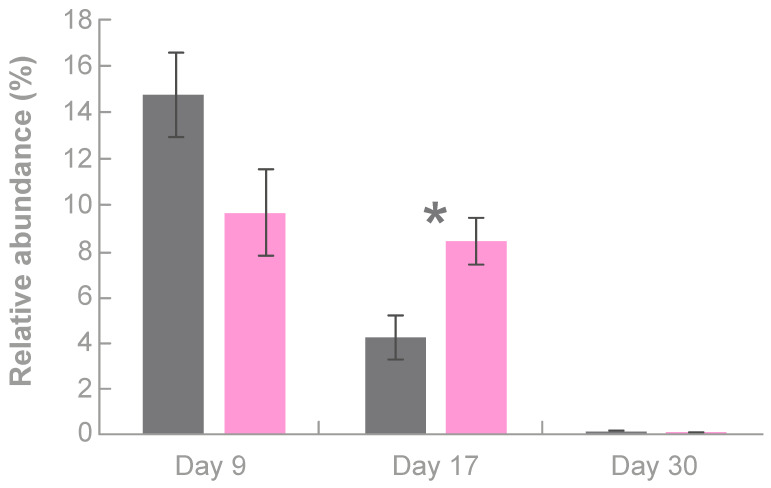
Relative abundance (%) of *Bacteroides* species in the gut of 9-, 17-, and 30-day-old piglets in the control (grey bars) and supplemented with Tonisity Px (pink bars) groups. Bars represent LSmeans and error bars denote ± SEM. * Indicates statistical significance between treatments at *p* < 0.05.

*Bacteroides* sp. abundance was not significantly different between groups at day 9 but, at day 17, members of this taxon were 1.9-fold more abundant in TPX piglets than in Control piglets (8.5% vs. 4.3%; SEM = 0.99; *p* < 0.05; Figure 3). *Bacteroides* sp. in the TPX group also stayed at a more consistent level at day 9 (9.6%) and day 17 (8.5%) compared to the Control group.

*Ruminococcus* unc01bm8, *Oscillospira* sp., and *Veillonella* sp. are less well-known beneficial bacteria but all were significantly more abundant in the TPX group than in the Control group at day 9. No differences were observed at day 17. *Ruminococcus* GHQCopro (*p* = 0.08) and *Veillonella* sp. (*p* < 0.01) were both increased at day 30.

#### 3.3.2. Effects of Tonisity Px on Potentially Pathogenic Bacteria

No treatment differences were observed for *Escherichia* sp. at any time point. The overall relative abundance of *Escherichia* sp. was 0.6% for both treatment groups (SEM = 0.27%). However, in both groups, *Escherichia* sp. was <1% at days 9 and 17, but increased sharply to >5.9% at day 30.

Pathogenic bacteria within genus *Actinobacillus*, order Clostridiales, and family Spirochaetaceae (which includes the known pig pathogen *Brachyspira hyodysenteriae*) showed lower relative abundances in pre-weaning TPX piglets than in pre-weaning Control piglets (Table 1 and Supplementary Table S3). At day 17, *Actinobacillus* sp. relative abundance in the TPX group (1.0%) was two times lower than in the Control group (2.2%) (SEM = 0.32; *p* = 0.09) but at day 30 an inverse relationship was found with *Actinobacillus* sp. being significantly more abundant in TPX than in Control piglets (1.2% vs. 0.4%; SEM = 0.14; *p* < 0.01). At day 9, the relative abundance of Spirochaetaceae was much lower in TPX than in Control piglets (0.04% vs. 0.15%; SEM = 0.02; *p* < 0.05) and a similar trend was recorded at day 17 for *Clostridiales UncCl291* (0.4% vs. 0.8%; SEM = 0.12 *p = 0.10*). *Helicobacter* sp. were only detected at low levels in some of the piglets, with a detection odds ratio of 4.3 lower in TPX piglets at day 9 (*p* = 0.08).

#### 3.3.3. Effects of Tonisity Px on Bacteria with Variable Roles

**Figure 4:**
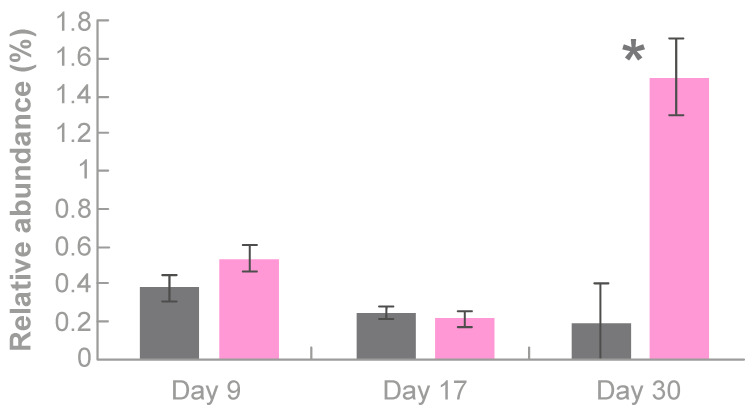
Relative abundance (%) of *Streptococcus* species in the gut of 9-, 17-, and 30-day-old piglets in the control (grey bars) and supplemented with Tonisity Px (pink bars) groups. Bars represent LSmeans and error bars denote ± SEM. * Indicates statistical significance between treatments at *p* < 0.05.

*Streptococcus* species have variable functional roles. As shown in Figure 4, a tendency for higher relative abundance of these species in TPX piglets than in Control piglets was observed at day 9 (0.5% vs. 0.4%; SEM = 0.05; *p* = 0.08), which became statistically-significant at day 30 (1.5 vs. 0.2%; SEM = 0.07; *p* < 0.01). *Romboutsia* sp. were also more abundant in TPX than in Control piglets at day 9 (0.9% vs. 0.5%; SEM = 0.13; *p* = 0.10).

*Prevotellaceae* show strain-specific roles and comprise 30–40% of all bacteria in the intestine. Its abundance was significantly lower in TPX piglets than in Control piglets at days 9 and 17 (27.6% vs. 37.0%; SEM = 2.15; *p* < 0.01). These differences represented a 29% reduction at day 9 and a 25% reduction at day 17, compared to Control piglets. However, no differences were observed at day 30.

### 3.4. Effects of Tonisity Px on E. coli and Rotavirus Incidence

In the pre-weaning phase (days 9 and 17 combined), the incidence of samples that were positive for *E. coli* was significantly lower in the TPX than in the Control group (26.9% vs. 58.3%; *p* = 0.05; Table 2). There was no significant difference in the incidence of *E. coli* between groups in the post-weaning phase (day 30), although it was still lower in TPX piglets (10%) compared to Control piglets (26.7%).

No significant difference in the incidence of RVA, RVB, or RVC was found between treatment groups at any age (Table 3).

## 4. Discussion

### 4.1. Pre-Weaning and Post-Weaning

Although it is known that the gut microbiota communities vary among individuals, and even among piglets within the same farming system, some general trends are known [12,16]. The general population results found in the present trial are congruent with previously published studies reporting Bacteroidetes and Firmicutes as some of the dominant phyla and *Prevotellaceae* and Ruminococcaceae as some of the dominant families [31,32]. The richness and diversity of the gut microbiota also tends to increase with age, particularly between pre-weaning and post-weaning [33]. In the present study, and regardless of dietary treatment, gut microbiota also significantly differed between pre-weaning and post-weaning piglets (i.e., at 9 and 17 days vs. 30 days).

Some of the main colonizers of the pre-weaning gut are *Lactobacillus sobrius*, *L. reuteri*, *L. acidophilus*, *E. coli*, and *Shigella flexneri*, but the relative abundances of *Lactobacillus* species drops significantly after weaning [34,35,36,37]. Compared to the control treatment, providing Tonisity Px to pre-weaned piglets increased the abundance of *Lactobacillus* species by 3.3-fold at day 9 and the abundance of *Bacteroides* species by 1.9-fold at day 17. An increase in *Lactobacillus* population is associated with improved gut maturation and digestion, as well as improved pathogen defense, better intestinal barrier function, and enhanced immunity [3,37]. A positive correlation between *Lactobacillus* species and growth performance has been confirmed in previous studies [3,38]. In the present study, supplementation with Tonisity Px stimulated the proliferation of *Lactobacillus* species, probably by delivering simple nutrients (carbohydrates, amino acids) to the enterocytes that *Lactobacilus* species can also use [39]. In addition, simple nutrients can encourage the growth of other bacterial groups, such as *Bacteroides* sp., that actively change the gut environment [40] and have been shown to improve gut function and to modulate the host immune system [40,41]. Although *Lactobacillus* and *Bacteroides* species both exert beneficial effects on their hosts, they usually have an inverse relationship with each other, as reported here (Figure 2) and in previous studies [42], likely because they compete for specific nutrients. This competition for specific nutrients could explain the higher abundance of *Lactobacillus* sp. than *Bacteroides* sp. in the TPX group at day 9 and the inverse relationship found at day 17.

Under natural conditions, weaning is a gradual process completed after 10–12 weeks but in production systems piglets are more commonly weaned after 3–4 weeks. This abrupt, early change occurs while the piglet’s digestive tract is experiencing a rapid period of expansion and specialization of the gut epithelium cells (enterocytes). These sudden dietary and environmental changes often result in reduced and irregular intake of feed and water (weaning anorexia) that contribute to gut inflammation, to reduced nutrient absorption and increased permeability of the gut epithelium to toxins and pathogens [43], and to a significant change in gut microbiota diversity, as reported here (Figure 1) and in previous studies [44]. A better pre-weaning microbial profile has been shown to minimize the consequences of weaning transition on pig performance post-weaning [10,18]. The results reported here support the use of the isotonic supplement because beneficial bacteria, such as *Lactobacillus* sp. and *Bacteroides* sp., showed a relative increase in pre-weaning TPX piglets (Figure 2) while some pathogenic bacteria, such as *Helicobacter* sp., Clostridiales, and Spirochaeteceae, decreased (Table 1).

### 4.2. E. coli and Pathogens

A major pathogen impacting the swine industry is *E. coli*, particularly enterotoxigenic *E. coli*, which is the main infectious agent of diarrhea in neonatal and post-weaning piglets. Supplementing Tonisity Px to piglets led to a 50% reduction in the percentage of piglets positive for *E. coli* in the pre-weaning phase. In addition, a higher number of intestinal *Lactobacillus* species has been linked to a reduction in *E. coli* [19]. Thus, the increase in *Lactobacillus* species abundance observed in the present study could be linked to the lower frequency of *E. coli* cultured from the ileum of piglets supplemented with Tonisity Px. Although piglets from the TPX group had a lower frequency of *E. coli* in the culture-based analyses (Section 3.4), this was not reflected in the microbial sequencing analysis (Section 3.3.2). This was most likely due to the sequencing analysis being performed at the genus level and not at the species/subspecies level.

With regards to *Streptococcus* species, their ability to cause pathology or act as beneficial bacteria is strain-specific. Intestinal streptococci have been used as probiotics in human and pig nutrition as they have been shown to have anti-inflammatory [45], antimicrobial [46], and immunostimulatory effects and are normal inhabitants of the digestive tract of pigs [47]. As a higher relative abundance of *Streptococcus* species was found in piglets supplemented with Tonisity Px and no pathology, either respiratory or intestinal, was detected in piglets from both study groups (Control and TPX), it is likely that the isotonic supplement enhanced the population of beneficial *Streptococcus* species.

### 4.3. Carbohydrate Digestion

In this study, during the pre-weaning period, *Ruminococcus* were increased and *Prevotellaceae* were decreased, which is in agreement with their preferential roles in degrading simple and complex carbohydrates, respectively. Similar to studies on human gut microbiota [42] and pig gut microbiota [1], *Prevotellaceae* and *Ruminococcus* species (belonging to order Clostridiales) were also inversely related in the present study. A further benefit of reducing *Prevotellaceae* in the pre-weaning period, when piglets are primarily on a milk-based diet, is that the *Prevotellaceae* can be scavengers. *Prevotellaceae* are known degraders of intestinal mucus and can destroy this important intestinal barrier [42]. A recent metagenomic analysis of the fecal microbiota in diarrheic piglets revealed an increase in the relative abundance of *Prevotella* species [34]. In the present study, the large decrease of *Prevotellaceae* in pre-weaning piglets supplemented with Tonisity Px supports the hypothesis of improved digestion provided by this isotonic protein solution. However, in the post-weaning period, when complex dietary carbohydrates are available, *Prevotellaceae* beneficially metabolize plant cell wall dietary fiber and produce significant amounts of short chain fatty acids that are later absorbed by the host [1]. These short-chain fatty acids also serve an anti-pathogen function [48,49]. The lack of difference in *Prevotellaceae* relative abundance between the TPX and Control groups post-weaning suggests that the fiber degrading capacity of the piglets was not affected by Tonisity Px, which therefore confers beneficial effects in the post-weaning period.

## 5. Conclusions

Tonisity Px supplied to piglets aged 2 to 8 days promoted a beneficial gut microbial profile by increasing the abundance of beneficial bacterial populations (e.g., *Lactobacillus* and *Bacteroides* species) and by reducing potentially pathogenic bacterial populations (e.g., *E. coli* and *Prevotellaceae*), while allowing the gut microbiota to adapt to nutrient availability. The protection provided by the *Lactobacillus* and *Bacteroides* species at days 9 and 17 of age, respectively, is likely to improve the gut environment for weaning. While the changes observed at days 9 and 17 could be linked to the nutritional effects of Tonisity Px on the enterocytes and beneficial bacterial populations, the changes occurring post-weaning, the most stressful time of the piglet’s life in terms of gut microbiota restructuring, are also worth noting. The increase and recovery of fiber-fermenting populations, along with the increase in *Veillonella* species, potential immune modulators, would be of interest for the long-term health of the intestine. Overall, the isotonic supplement tested here modulated the gut of piglets so as to increase the abundance of beneficial bacterial populations and reduce the potentially pathogenic ones, in both the pre-weaning and post-weaning periods.

## Figures and Tables

**Table 1:** Changes in the relative abundance of bacterial taxa in response to Tonisity Px supplementation. Representative species within each observed taxa are presented in parentheses.

Change	Beneficial	Potentially Pathogenic	Variable Role
Increased	*Lactobacillus*	*Actinobacillus (A. pleuropneumoniae)* post-weaning	*Streptococcus (S. salivarius thermophilus)* *Romboutsia*
	*(L. acidophilus)*		
	*Bacteroides*		
	*(B. fragilis)*		
	*Ruminococcus*		
	*(R. flavefaciens)*		
	*Oscillospira*		
	*(O. guillermondii)*		
	*Veillonella*		
	*(V. parvula)*		
Decreased		*Actinobacillus*	*Prevotellaceae (P. ruminicola)*
		*(A. pleuropneumoniae)*	
		pre-weaning	
		Clostridiales	
		*(C. perfringens)*	
		Spirochaetaceae	
		*(Brachyspira hyodisenteriae)*	
		*Helicobacter*	
		*(H. pylori)*	
Not affected		*Escherichia (E. coli)*	

**Table 2:** Incidence of culture samples positive for *E. coli* in pre-weaning and post-weaning piglets in the TPX and Control groups.

Production Phase	Treatment	Unit	*E. coli*		Total *n*	*p* *
			Negative	Positive		
Pre-Weaning	Control	*n*	12	14	26	0.05
		*%*	46.2%	53.8%		
	TPX	*n*	19	7	26	
		*%*	73.1%	26.9%		
	Total		31	21		
Post-Weaning	Control	*n*	9	1	10	0.16
		*%*	90%	10%		
	TPX	*n*	5	3	8	
		*%*	62.5%	37.5%		
	Total		14	4		

* Calculated using the Chi-square test in SAS 9.4.

**Table 3:** Incidence (number and %) of RVA, RVB, and RVC positive samples in the TPX and Control groups.

*Rotavirus* Serogroup	Control (%)	TPX (%)	p *
RVA	12/36 (33.3%)	10/40 (25%)	0.424
RVB	10/36 (27.8%)	8/39 (20.5%)	0.462
RVC	3/35 (8.57%)	6/39 (15.4%)	0.486

* Calculated using the Chi-square test in SAS 9.4.

## Supplementary Materials

The following are available online at https://www.mdpi.com/2076-2615/10/5/879/s1, Table S1: Gilt lactation diet. Table S2: Piglet creep feed diet. Table S3: Changes in the relative abundance of beneficial, potentially pathogenic, and variable-role bacteria in the gut of 9-, 17-, and 30-day-old piglets in the Control and TPX groups.

## References

[1] Ramayo-Caldas Y., Mach N., Lepage P., Levenez F., Denis C., Lemonnier G., Leplat J.-J., Billon Y., Berri M., Doré J. (2016). Phylogenetic network analysis applied to pig gut microbiota identifies an ecosystem structure linked with growth traits. ISME J..

[2] Gensollen T., Iyer S.S., Kasper D.L., Blumberg R.S. (2016). How colonization by microbiota in early life shapes the immune system. Science.

[3] Cheng C., Wei H., Xu C., Xie X., Jiang S., Peng J. (2018). Maternal soluble fiber diet during pregnancy changes the intestinal microbiota, improves growth performance, and reduces intestinal permeability in piglets. Appl. Environ. Microbiol..

[4] Jayaraman B., Nyachoti C.M. (2017). Husbandry practices and gut health outcomes in weaned piglets: A review. Anim. Nutr..

[5] Pluske J.R., Turpin D.L., Kim J.-C. (2018). Gastrointestinal tract (gut) health in the young pig. Anim. Nutr..

[6] Everaert N., Van Cruchten S., Weström B., Bailey M., Van Ginneken C., Thymann T., Pieper R. (2017). A review on early gut maturation and colonization in pigs, including biological and dietary factors affecting gut homeostasis. Anim. Feed Sci. Technol..

[7] Mulder I.E., Schmidt B., Lewis M., Delday M., Stokes C.R., Bailey M., Aminov R.I., Gill B.P., Pluske J.R., Mayer C.-D. (2011). Restricting microbial exposure in early life negates the immune benefits associated with gut colonization in environments of high microbial diversity. PLoS ONE.

[8] Mulder I.E., Schmidt B., Stokes C.R., Lewis M., Bailey M., Aminov R.I., Prosser J.I., Gill B.P., Pluske J.R., Mayer C.-D. (2009). Environmentally-acquired bacteria influence microbial diversity and natural innate immune responses at gut surfaces. BMC Biol..

[9] Schmidt B., Mulder I.E., Musk C.C., Aminov R.I., Lewis M., Stokes C.R., Bailey M., Prosser J.I., Gill B.P., Pluske J.R. (2011). Establishment of normal gut microbiota is compromised under excessive hygiene conditions. PLoS ONE.

[10] Cheng C., Wei H., Wang P., Yu H., Zhang X., Jiang S., Peng J. (2019). Early intervention with faecal microbiota transplantation: An effective means to improve growth performance and the intestinal development of suckling piglets. Animal.

[11] Liao S.F., Nyachoti M. (2017). Using probiotics to improve swine gut health and nutrient utilization. Anim. Nutr..

[12] Isaacson R., Kim H.B. (2012). The intestinal microbiome of the pig. Anim. Health Res. Rev..

[13] Zeineldin M., Aldridge B., Lowe J. (2018). Dysbiosis of the fecal microbiota in feedlot cattle with hemorrhagic diarrhea. Microb. Pathog..

[14] Heo J., Opapeju F., Pluske J., Kim J., Hampson D., Nyachoti C. (2013). Gastrointestinal health and function in weaned pigs: A review of feeding strategies to control post-weaning diarrhoea without using in-feed antimicrobial compounds. J. Anim. Physiol. Anim. Nutr..

[15] Guarner F., Malagelada J.-R. (2003). Gut flora in health and disease. Lancet.

[16] Kim H.B., Isaacson R.E. (2015). The pig gut microbial diversity: Understanding the pig gut microbial ecology through the next generation high throughput sequencing. Vet. Microbiol..

[17] Zhao Y., Su J.-Q., An X.-L., Huang F.-Y., Rensing C., Brandt K.K., Zhu Y.-G. (2018). Feed additives shift gut microbiota and enrich antibiotic resistance in swine gut. Sci. Total Environ..

[18] Guevarra R.B., Lee J.H., Lee S.H., Seok M.-J., Kim D.W., Kang B.N., Johnson T.J., Isaacson R.E., Kim H.B. (2019). Piglet gut microbial shifts early in life: Causes and effects. J. Anim. Sci. Biotechnol..

[19] Gresse R., Chaucheyras-Durand F., Fleury M.A., Van de Wiele T., Forano E., Blanquet-Diot S. (2017). Gut microbiota dysbiosis in postweaning piglets: Understanding the keys to health. Trends Microbiol..

[20] Hakim R.S., Baldwin K., Smagghe G. (2010). Regulation of midgut growth, development, and metamorphosis. Annu. Rev. Entomol..

[21] Firth A., Martin R., Cano G. Effect of Tonisity Px administration on pre-weaning mortality and weight gain. Proceedings of the 48th AASV Annual Meeting.

[22] Firth A., Cano G. Effect of gruel and Tonisity Px on feed intake and weight gain at weaning. Proceedings of the 48th AASV Annual Meeting.

[23] Caporaso J.G., Lauber C.L., Walters W.A., Berg-Lyons D., Huntley J., Fierer N., Owens S.M., Betley J., Fraser L., Bauer M. (2012). Ultra-high-throughput microbial community analysis on the Illumina HiSeq and MiSeq platforms. ISME J..

[24] Edgar R.C. (2010). Search and clustering orders of magnitude faster than BLAST. Bioinformatics.

[25] Edgar R.C. (2013). UPARSE: Highly accurate OTU sequences from microbial amplicon reads. Nat. Methods.

[26] Quast C., Pruesse E., Yilmaz P., Gerken J., Schweer T., Yarza P., Peplies J., Glöckner F.O. (2012). The SILVA ribosomal RNA gene database project: Improved data processing and web-based tools. Nucleic Acids Res..

[27] Lozupone C., Knight R. (2005). UniFrac: A new phylogenetic method for comparing microbial communities. Appl. Environ. Microbiol..

[28] (2019). R: A Language and Environment for Statistical Computing.

[29] Oksanen J., Kindt R., Legendre P., O’Hara B., Stevens M.H.H., Oksanen M.J., Suggests M. (2007). The vegan package. Community Ecol. Package.

[30] Marthaler D., Homwong N., Rossow K., Culhane M., Goyal S., Collins J., Matthijnssens J., Ciarlet M. (2014). Rapid detection and high occurrence of porcine rotavirus A, B, and C by RT-qPCR in diagnostic samples. J. Virol. Methods.

[31] Buzoianu S.G., Walsh M.C., Rea M.C., O’Sullivan O., Cotter P.D., Ross R.P., Gardiner G.E., Lawlor P.G. (2012). High-throughput sequence-based analysis of the intestinal microbiota of weanling pigs fed genetically modified MON810 maize expressing Bacillus thuringiensis Cry1Ab (Bt maize) for 31 days. Appl. Environ. Microbiol..

[32] Buzoianu S.G., Walsh M.C., Rea M.C., O’Sullivan O., Crispie F., Cotter P.D., Ross R.P., Gardiner G.E., Lawlor P.G. (2012). The effect of feeding Bt MON810 maize to pigs for 110 days on intestinal microbiota. PLoS ONE.

[33] Buzoianu S.G., Walsh M.C., Rea M.C., Quigley L., O’Sullivan O., Cotter P.D., Ross R.P., Gardiner G.E., Lawlor P.G. (2013). Sequence-based analysis of the intestinal microbiota of sows and their offspring fed genetically modified maize expressing a truncated form of Bacillus thuringiensis Cry1Ab protein (Bt maize). Appl. Environ. Microbiol..

[34] Lallès J.-P., Bosi P., Smidt H., Stokes C.R. (2007). Weaning—A challenge to gut physiologists. Livest. Sci..

[35] Konstantinov S.R., Zhu W.-Y., Williams B.A., Tamminga S., de Vos W.M., Akkermans A.D. (2003). Effect of fermentable carbohydrates on piglet faecal bacterial communities as revealed by denaturing gradient gel electrophoresis analysis of 16S ribosomal DNA. FEMS Microbiol. Ecol..

[36] Konstantinov S.R., Awati A.A., Williams B.A., Miller B.G., Jones P., Stokes C.R., Akkermans A.D., Smidt H., De Vos W.M. (2006). Post-natal development of the porcine microbiota composition and activities. Environ. Microbiol..

[37] Inoue R., Tsukahara T., Nakanishi N., Ushida K. (2005). Development of the intestinal microbiota in the piglet. J. Gen. Appl. Microbiol..

[38] Hou C., Zeng X., Yang F., Liu H., Qiao S. (2015). Study and use of the probiotic Lactobacillus reuteri in pigs: A review. J. Anim. Sci. Biotechnol..

[39] Walter J. (2008). Ecological role of lactobacilli in the gastrointestinal tract: Implications for fundamental and biomedical research. Appl. Environ. Microbiol..

[40] Wexler A.G., Goodman A.L. (2017). An insider’s perspective: Bacteroides as a window into the microbiome. Nat. Microbiol..

[41] Wexler H.M. (2007). Bacteroides: The good, the bad, and the nitty-gritty. Clin. Microbiol. Rev..

[42] Arumugam M., Raes J., Pelletier E., Le Paslier D., Yamada T., Mende D.R., Fernandes G.R., Tap J., Bruls T., Batto J.-M. (2011). Enterotypes of the human gut microbiome. Nature.

[43] Barton M.D. (2014). Impact of antibiotic use in the swine industry. Curr. Opin. Microbiol..

[44] Lalles J.-P., Bosi P., Smidt H., Stokes C.R. (2007). Nutritional management of gut health in pigs around weaning. Proc. Nutr. Soc..

[45] Del Carmen S., de LeBlanc A.d.M., Martin R., Chain F., Langella P., Bermúdez-Humarán L.G., LeBlanc J.G. (2014). Genetically engineered immunomodulatory Streptococcus thermophilus strains producing antioxidant enzymes exhibit enhanced anti-inflammatory activities. Appl. Environ. Microbiol..

[46] O’Shea E.F., Gardiner G.E., O’Connor P.M., Mills S., Ross R.P., Hill C. (2009). Characterization of enterocin-and salivaricin-producing lactic acid bacteria from the mammalian gastrointestinal tract. FEMS Microbiol. Lett..

[47] Goyette-Desjardins G., Auger J.-P., Xu J., Segura M., Gottschalk M. (2014). Streptococcus suis, an important pig pathogen and emerging zoonotic agent—An update on the worldwide distribution based on serotyping and sequence typing. Emerg. Microbes Infect..

[48] Liu B., Wang W., Zhu X., Sun X., Xiao J., Li D., Cui Y., Wang C., Shi Y. (2018). Response of gut microbiota to dietary fiber and metabolic interaction with SCFAs in piglets. Front. Microbiol..

[49] Foo J.L., Ling H., Lee Y.S., Chang M.W. (2017). Microbiome engineering: Current applications and its future. Biotechnol. J..

