# Supplementary material for: Early-Life Intake of an Isotonic Protein Drink Improves the Gut Microbial Profile of Piglets
Source: Animals (Basel). 2020 May 18;10(5):879. doi: 10.3390/ani10050879 (PMC7278368; doi:10.3390/ani10050879)
Supplement: Supplementary file 1 [file animals-10-00879-s001.pdf]

Table 1. Gilt lactation diet.

	Units	Actual	Dry matter
Weight	Lb	1.0000	1.0000
Moisture	%	10.9129	12.2497
dLYS:Ne	g/Mcal	2.3447	2.3447
ME swine	Kcal/Lb	1 449.17	1 626.69
NE swine	Kcal/Lb	1 104.805	1 240.140
Crude Protein	%	12.4838	14.0130
Lysine	%	0.6725	0.7549
SSID Lysine	%	0.5711	0.6411
MET+CYS	%	0.4847	0.5441
Crude Fat	%	5.0275	5.6433
Crude Fiber	%	5.1383	5.7678
Calcium	%	0.8128	0.9124
Phosphorus	%	0.6314	0.7088
STTDig PHOS SW	%	0.5685	0.6381
Salt	%	0.5000	0.5612
Sodium	%	0.2729	0.3063
Chloride	%	0.5004	0.5617
Copper	ppm	26.0424	29.2325
Iodine	ppm	0.9581	1 0755
Iron	ppm	162.5155	182.4231
Manganese	ppm	72.5998	81.4930
Selenium	ppm	0.3000	0.3367
Zinc	ppm	140.6008	157.8239
Vitamin A	KIU/Lb	6.0045	6.7400
Vitamin D	KIU/Lb	1.1507	1.2917
Vitamin E	IU/Lb	39.1775	43.9766
Vit K/Menadione	mg/Lb	2.1382	2.4001
Biotin	mg/Lb	0.2002	0.2247
Vitamin B12	mg/Lb	0.0202	0.0227
Choline	mg/Lb	677.7031	760.7194
Folic Acid	mg/Lb	1.6000	1.7960
Niacin	mg/Lb	22.5150	25.2730
Pantothenic Acid	mg/Lb	15.0105	16.8493
Pyridoxine	mg/Lb	2.2511	2.5269
Riboflavin	mg/Lb	4.4021	4.9413
Thiamine	mg/Lb	1.0016	1.1243
Phytase Units	Flu/Lb	1,183.9	1,328.9
dlLE/dLYS		0.6283	0.6283
dM+C/dLYS		0.7243	0.7243
dTHR/dLYS		0.7675	0.7675
dTRP/dLYS		0.1735	0.1735
dVAU/dLYS		0.8445	0.8445

Table 2. Piglet creep diet.

Formula Code	Description		
10605212	BIP Phase 1 N-M		
**Code**	**Ingredient**	**Amount**	**Pct**
91000101	Corn 6.9%	825.7732	41.29
92022101	SBM 46%	540.5667	27.03
91106102	Lactose FG	163.2651	8.16
91047101	Breakfast Cereal Fines BU	100.0000	5.00
91072101	Feeding Oatmeal Bulk	100.0000	5.00
92019101	Corn Dog W/Sol Wint	75.0000	3.75
91001701	Animal Fat/Choice White	60.0000	3.00
92013101	Fish Meal/IPC 790	60.0000	3.00
93120102	PHOS/Monocal 22.7% P	18.3900	0.92
93014101	Calcium Carb 39%	14.3880	0.72
99033102	DPX 9902	10.0000	0.50
94042102	Zinc Oxide 72%	8.1339	0.41
93006102	Salt/Mix & Fines	8.0000	0.40
99024172	L-Lysine 99%	5.6211	0.28
70113192	Swine Starter Vtm Pmx	2.5000	0.13
99011172	Dl-Meth 99%	2.2029	0.11
95008102	Choline Chlor 60%	1.6000	0.08
99023170	L-Threonine 98.5%	1.5031	0.08
96021771	Flav/Carmel Liquid	1.0000	0.05
11247103	Tm Swine Tm (no Se)	0.7352	0.04
94000172	Intellibond C Tb Cu Cl	0.5882	0.03
12665132	Phytase 10 000	0.3000	0.02
94023103	ALKOSEL 3 000 (Se Yeast)	0.2000	0.01
99009175	L-Tryptophan 98%	0.1327	0.01
94011172	Chromium Propionate 0.4%	0.1000	

	Units	Actual	Dry matter
Weight	*Lb*	*1.0000*	*1.0000*
Moisture	*%*	*10.3050*	*11.4889*
dLYS:Ne	*g/Mcal*	*5.0193*	*5.0193*
ME swine	*Kcal/Lb*	*1 561.39*	*1 740.77*
NE swine	*Kcal/Lb*	*1 145.278*	*1 276.858*
Sugar	%	137.6328	153.4453
Crude Protein	%	19.6452	21.9022
Lysine	%	1.3872	1.5466
SSID Lysine	%	1.2673	1.4129
MET+CYS	%	0.7742	0.8632
Crude Fat	%	6.0933	6.7933
Crude Fiber	%	2.4005	2.6763
Lactose	%	8.0000	8.9191
Calcium	%	0.6938	0.7735
Phosphorus	%	0.6500	0.7247

STTDig PHOS SW	%	0.5164	0.5757
Salt	%	0.4500	0.5017
Sodium	%	0.2220	0.2475
Chloride	%	0.3940	0.4393
Copper	ppm	200.6489	223.7013
Iodine	ppm	1.6212	1.8075
Iron	ppm	161.9701	180.5786
Manganese	ppm	73.8261	82.3079
Selenium	ppm	0.3000	0.3345
Zinc	ppm	3 048.7400	3 399.0070
Vitamin A	KIU/Lb	6.0013	6.6907
Vitamin D	KIU/Lb	1.1018	1.2283
Vitamin E	IU/Lb	56.2939	62.7615
Vit K/Menadione	mg/Lb	4.4445	4.9551
Biotin	mg/Lb	0.1588	0.1770
Vitamin B12	mg/Lb	0.0298	0.0332
Choline	mg/Lb	730.5539	814.4865
Folic Acid	mg/Lb	1.0905	1.2158
Niacin	mg/Lb	32.6563	36.4081
Pantothenic Acid	mg/Lb	20.7563	23.1409
Pyridoxine	mg/Lb	4.4443	4.9549
Riboflavin	mg/Lb	6.3100	7.0350
Thiamine	mg/Lb	3.3331	3.7160
Phytase Units	flu/Lb	680.5	758.6
dlLE/dLYS		0.6170	0.6170
dM+C/dLYS		0.5538	0.5538
dTHR/dLYS		0.5919	0.5919
dTRP/dLYS		0.1709	0.1709
dVAU/dLYS		0.6777	0.6777
Live Microbial	MIU/g	1.106	1.233

Table S3. Changes in the relative abundance of beneficial, potentially pathogenic, and variable-role bacteria in the gut of 9-, 17-, and 30-day-old piglets in the Control and TPX groups.

Taxon	Day	Control	TPX	SEM	*P* value		
					Treatment × Day	Overall Treatment	Day
Beneficial bacteria							
	9	1.2	4.0	0.29	<0.0001		
Lactobacillus	17	1.5	2.0	0.23	0.27	0.13	<0.0001
	30	2.0	1.4	0.23	0.21		
	9	14.8	9.6	1.85	0.17		
Bacteroides	17	4.3	8.5	0.99	0.04	0.66	<0.0001
	30	0.04	0.02	0.01	0.11		
	9	1.4	2.0	0.33	0.40		
Ruminococcaceae GHQCopro	17	0.9	1.2	0.21	0.42	0.24	<0.0001
	30	0.3	0.6	0.08	0.08		
	9	0.2	0.6	0.10	0.05		
Ruminococcaceae Unc01bm8	17	3.0	2.5	0.73	0.76	0.74	<0.0001
	30	0.2	0.2	0.05	0.52		
	9	0.2	0.7	0.08	0.01		
Oscillospira	17	0.5	0.7	0.13	0.35	0.44	<0.0001
	30	0.2	0.1	0.04	0.19		
	9	0.8	1.5	0.16	0.03		
Veillonella	17	0.6	0.4	0.07	0.27	0.01	<0.0001
	30	0.2	1.4	0.08	0.00		
Potentially-pathogenic bacteria							
	9	3.4	3.1	0.67	0.82		
Actinobacillus	17	2.2	1.0	0.32	0.09	0.79	<0.0001
	30	0.4	1.2	0.14	0.01		
	9	0.4	0.6	0.11	0.50		
Clostridiales_UncCl291	17	0.8	0.4	0.12	0.10	0.86	<0.0001
	30	0.2	0.4	0.06	0.17		
	9	0.15	0.04	0.02	0.02		
Spirochetaceae	17	0.36	0.31	0.09	0.78	0.27	<0.0001
	30	0.54	0.40	0.13	0.56		
	9	0.8	0.3	0.16	0.21		
Escherichia	17	0.1	0.1	0.02	0.88	0.90	<0.0001
	30	5.9	9.0	2.18	0.48		
Bacteria with variable roles							
	9	0.38	0.54	0.05	0.08		
Streptococcus	17	0.25	0.22	0.03	0.50	0.0002	<0.0001
	30	0.20	1.5	0.07	<0.0001		
	9	0.5	0.9	0.13	0.10		
Romboutsia	17	1.1	0.6	0.15	0.15	0.90	<0.0001
	30	0.1	0.1	0.03	0.88		
	9	29.0	20.5	1.80	0.002		
Prevotellaceae	17	37.0	27.6	2.15	0.003	0.04	<0.0001
	30	44.3	44.4	2.50	0.99		

Statistical significance between treatments at $P < 0.05$ was evaluated using the generalized mixed model within the GLIMMIX procedure of SAS 9.4.
